# Hospital Weakness and Hospital Strength

**Published:** 1913-03-01

**Authors:** 


### HOSPITAL WEAKNESS AND HOSPITAL STRENGTH.

(Contributions to this page will be welcomed.)

### Some Committees which Fail: Two Amazing Examples.

We have had occasion in this department of the paper to speak quite straightly about various people, high and low, who may fail to be efficient for the reasons which have been distinctly given, and whose retirement would constitute an asset of value to the administrative efficiency of the institution to which they may be attached. Government by committee under the voluntary system, where the standard of personal conduct is high and is continuously maintained through the enthusiastic example of a leader or leaders in the person of the chairman or treasurer or other high official, has proved in this country to be better, on the whole, than any other system. Indeed, the great value of the voluntary system is that it attracts men of the highest character and commanding ability from a love of the work to give their time and best brains to the administration of many of our most important hospitals and kindred institutions. Forty years ago, and previous to that date, the merits of this system of government were discounted by some grave defects. These defects were largely due to patronage centred in the hands of a few individuals or families who had inherited associations with particular charities and so became members of committee. As the importance of the work of the voluntary hospitals has increased and developed the cost of maintaining these institutions has enormously grown, with the result that the area of givers may have grown in some proportion to the needs of each such institution. In this way the governors have continually increased in numbers until these old abuses have almost everywhere died a natural death.

### Superannuation and Age Limits.

In these days our modern hospitals worked on the voluntary system are recognised to be, on the whole, the most efficient in the world. It would at first sight appear to be well-nigh impossible that the committee or any sub-committee of such an institution could degenerate into grave inefficiency. Yet to our regret we find that a few such cases do exist in connection with voluntary hospitals in various parts of the country. Having regard to the nature and responsibilities attaching to hospital work in the present day, such inefficiency is a grave scandal, and may become a cruelty to the patients and a wanton abuse of the privileges of trusteeship which devolve upon members of a hospital committee. Sometimes an official highly placed is allowed to retain office after he has reached the limit of threescore years and ten, and by virtue of his long connection may regard himself as indispensable and to possess the right of posing as the master of the whole establishment, though he may be and often is gravely incapable of fulfilling the duties of such a position. Under such a man a committee is apt to lose its vitality and to exist rather in name than in fact. We inspected an incurable hospital the other day in the North of England where such a state of affairs exists, and where in consequence the whole atmosphere of the establishment has degenerated, to the great discomfort of the more respectable type of patient and to the degeneracy of those of a different type. If the actual condition of this institution and the abuses which exist in it at the present time are ever published they will create a storm which is calculated to bring into contempt the name of every man and woman associated with the management at the present time.

### Family Cliques.

Another dangerous type of committee is that where the dominant spirits among its members consist of an ex-official—a matron, for instance—who may have married a member of the medical staff, where two such individuals hob-nob with or control the matron of to-day and so prevent radical changes from being introduced to safeguard the interests of the patients and to protect the nursing staff from abuses which may amount to cruelty in practice by their continuance. We found this state of affairs in a hospital not long ago, and inferentially reported upon it, feeling called upon seriously to warn the governors and the committee of the actual state of affairs within this hospital. That warning has produced no effect in the way of improvement, change, or reform. It was met, we are advised, by a statement from the secretary that the writer of the report had no knowledge of hospital administration, and that the warning was uncalled for and even impertinent. A year has elapsed since then, in which it has become abundantly apparent that every probationer who is engaged at this institution could probably, if she chose, bring an action for damages against the management on the ground that she was not being trained properly or systematically as a nurse. The friends of one of the nurses at least, who has since died under circumstances of appalling neglect and gross mismanagement, might recover damages if they could not even sustain an action for manslaughter against the matron and the house committee; just as onerous is the responsibility of the medical staff who have known the facts and still permit these grave evils to continue without redress. The case of the patients in such conditions may be hard indeed. We venture to think that in the history of voluntary hospitals there has probably never been a children's ward containing a larger number of severe cases where the neglect and the resulting cruelties have been anything like so grave as those in the institution we refer to.

A state of affairs like that we have just instanced must have grave results, for if continued it must ensure that the deans of the principal medical schools and universities throughout the country will be obliged in self-defence to advise their graduates and qualified men not to apply for the resident medical posts in such an institution. We may have more to say in regard to this matter on another occasion. It may be well to add that our knowledge of the precise accuracy of the forecast which we made in our report published in The Hospital about a year ago is due to the advice and action of one of the professors of a great university who was consulted on the subject by two of his graduates, who were appalled as resident medical officers at the state of affairs which they found to exist at the hospital in question.

